# The genome sequence of the Sandhill Rustic moth *Luperina nickerlii *(Freyer, 1845) subspecies *leechi* Goater, 1976

**DOI:** 10.12688/wellcomeopenres.22623.1

**Published:** 2024-07-17

**Authors:** Adrian Spalding, Walther Traut, Richard H. ffrench-Constant, Natural History Museum Genome Acquisition Lab, Darwin Tree of Life Barcoding collective, Wellcome Sanger Institute Tree of Life Management, Samples and Laboratory team, Wellcome Sanger Institute Scientific Operations: Sequencing Operations, Wellcome Sanger Institute Tree of Life Core Informatics team, Tree of Life Core Informatics collective, Darwin Tree of Life Consortium

**Affiliations:** 1 University of Exeter, Tremough, Penryn, England, UK; 2 Institut for Biology, University of Lubeck, Lubeck, Germany

**Keywords:** Luperina nickerlii leechi, Sandhill Rustic, genome sequence, chromosomal, Lepidoptera

## Abstract

We present a genome assembly from an individual female *Luperina nickerlii* (the Sandhill Rustic; Arthropoda; Insecta; Lepidoptera; Noctuidae). The genome sequence is 662.0 megabases in span. Most of the assembly is scaffolded into 31 chromosomal pseudomolecules, including the Z sex chromosome. The specimen was confirmed to be a ZO female. The mitochondrial genome has also been assembled and is 15.47 kilobases in length.

## Species taxonomy

Eukaryota; Metazoa; Ecdysozoa; Panarthropoda; Arthropoda; Hexapoda; Insecta; Pterygota; Neoptera; Endopterygota; Lepidoptera; Glossata; Neolepidoptera; Ditrysia; Noctuoidea; Xyleninae; *Luperina*; *Luperina nickerlii* (Freyer 1845); *Luperina nickerlii leechi* Goater, 1976 (NCBI:txid882792).

## Background

The Sandhill Rustic moth *Luperina nickerlii* (Freyer, 1845) is an Atlantico-Mediterranean species of nocturnal moth not found outside Europe. It has been recorded from across western Europe in the Czech Republic (where it was first found), Andorra, Bosnia and Herzogovia, Croatia, France, Germany, Great Britain, Ireland, Italy, Kosovo, Macedonia, Montenegro, Poland, Portugal, Slovenia and Spain. It has been found from sea level up to the heights of the Pyrenees at 1,950 m. Britain supports the northernmost colonies of this moth, which is rare in northern continental Europe but found in extensive populations in the southern hills and mountains.

There are several subspecies, the precise status of which is still open to debate ( Spalding, 2015). However, the following subspecies have so far been identified:

- *nickerlii* Freyer, 1845, found on sandy heaths throughout Germany and France where the larvae feed on *Festuca ovina*
- *graslini* Oberthür, 1908, found on limestone hills in France where the larvae feed on *Festuca ovina*
- *tardenota* Joannis, 1925 found in a small area south of Paris where the larvae feed on *Festuca ovina*
- *albarracina* Schwingenschuss, 1962 found on hot dry slopes in Spain and Portugal where the larvae feed on *Festuca rubra* and *Festuca ovina*
- *knilli* Boursin, 1964 found on cliffs south-west Ireland where the larvae feed on *Festuca rubra*
- *gueneei* Doubleday, 1864 found on sand dunes in north Wales and Lancashire in the UK where the larvae feed on *Eytrigia juncea*
- *demuthi* Goater & Skinner 1995 found in saltmarshes in Essex, Kent and Suffolk in the UK where the larvae feed on *Puccinellia maritima*
- *leechi* Goater, 1976 found on a single sand and shingle beach in Cornwall in south-west England where the larvae feed on *Elytrigia jucea*.

Subspecies *tardenota*, *gueneei* and especially *leechi* have small populations in a restricted area and are potentially under threat from habitat loss and climate change. Subspecies *demuthi* is unique in that it can survive under brackish water. Adult moths fly in late August through to October and will come to light. The coastal sub-species are weak fliers and will only fly on nights with light or no winds. Eggs are laid in parallel rows inside the sheaths of the larval foodplant. Larvae overwinter inside the grass stem and then in spring feed inside the grass crowns or underground on the rhizomes and roots. Pupae usually have a silky case around them.

The adult moth is visually similar to *Luperina testacea* but may generally be distinguished by its silky forewing, small round orbicular stigma and especially by the white edge to the reniform stigma. Subspecies *leechi* and *gueneei* are clearly different from *testacea* being much lighter in colour, but other subspecies can be difficult to separate from *testacea* in the field, especially when the edge to the reniform stigma is pale rather than white. There are small differences in the genitalia. Differences in the female genitalia are very slight, only apparent in the antrum or ostium bursae (the opening in the genital plate) sclerotized band which is 5 times broader than high in *nickerlii* and slightly higher in *testacea*. The main differences in the male genitalia lie in the ventral tip of the valve head, which is more rounded in *nickerlii*, the angle of the clavus apex which is more prominent and the aedeagus which has more spines.

**Figure 1.**
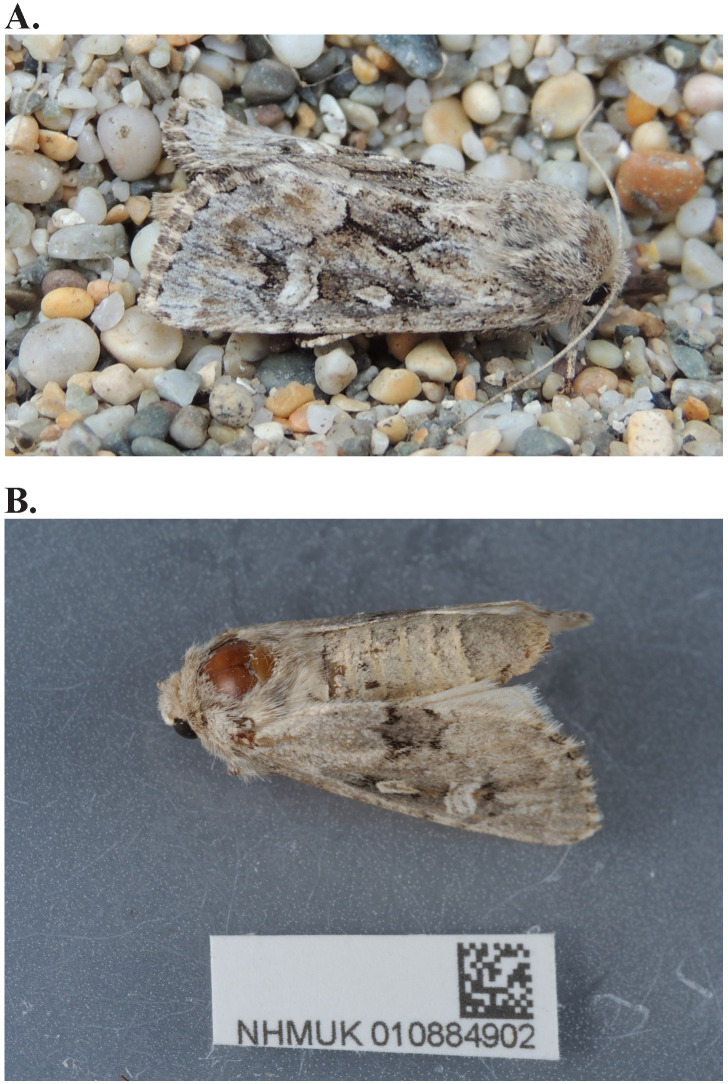
**A**. *Luperina nickerlii leechi* photographed on Loe Bar, Cornwall showing cryptic colouration (photo by Adrian Spalding), **B**. Photograph of the *Luperina nickerlii* (ilLupNick1) specimen used for genome sequencing.

*Luperina nickerlii leechi* occupies a unique isolated population at least 300 km from the nearest other *nickerlii* populations and exists on a small sand / shingle bar just 430 m long and 250 m wide, Loe Bar near Helston in Cornwall. The adult moth is uniquely coloured with pale silver and brown forewings which match the colours of the mixed pebbly shingle substrate ( Figure 1). The population of *leechi* is unusual in that the male : female ratio has been found to be 30:70, rather than the expected 50 : 50 sex ratio more commonly found in Lepidoptera ( Clarke, 1984).

## Genome sequence report

The genome was sequenced from an adult female *Luperina nickerlii* ( Figure 1) collected from Penrose Estate, Loe Bar, England, UK (50.08, –5.29). A total of 59-fold coverage in Pacific Biosciences single-molecule HiFi long reads was generated. Primary assembly contigs were scaffolded with chromosome conformation Hi-C data. Manual assembly curation corrected 6 missing joins or mis-joins.

**Figure 2.**
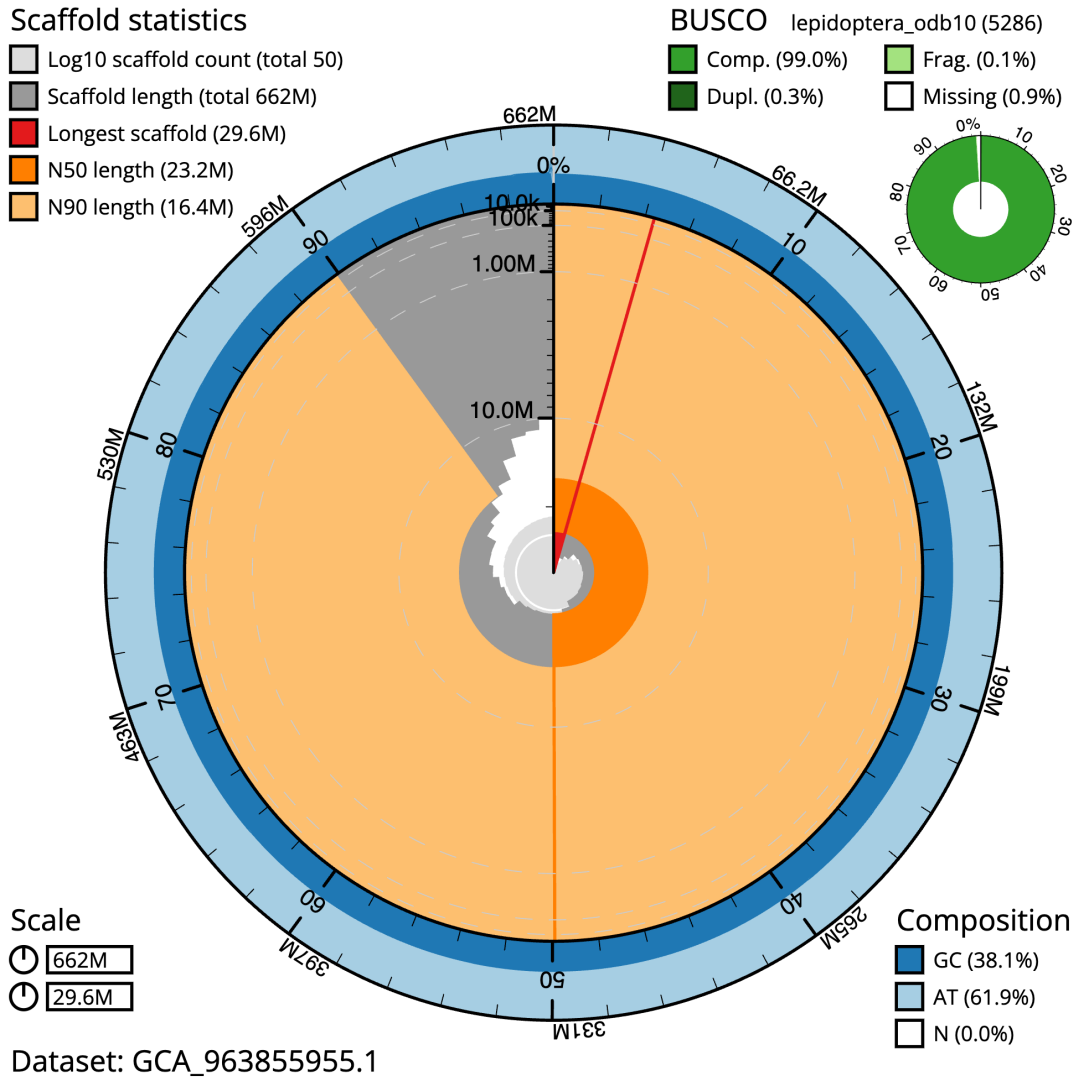
Genome assembly of *Luperina nickerlii*, ilLupNick1.1: metrics. The BlobToolKit snail plot shows N50 metrics and BUSCO gene completeness. The main plot is divided into 1,000 size-ordered bins around the circumference with each bin representing 0.1% of the 661,975,145 bp assembly. The distribution of scaffold lengths is shown in dark grey with the plot radius scaled to the longest scaffold present in the assembly (29,637,245 bp, shown in red). Orange and pale-orange arcs show the N50 and N90 scaffold lengths (23,242,021 and 16,383,867 bp), respectively. The pale grey spiral shows the cumulative scaffold count on a log scale with white scale lines showing successive orders of magnitude. The blue and pale-blue area around the outside of the plot shows the distribution of GC, AT and N percentages in the same bins as the inner plot. A summary of complete, fragmented, duplicated and missing BUSCO genes in the lepidoptera_odb10 set is shown in the top right. An interactive version of this figure is available at https://blobtoolkit.genomehubs.org/view/Luperina_nickerlii/dataset/GCA_963855955.1/snail.

**Figure 3.**
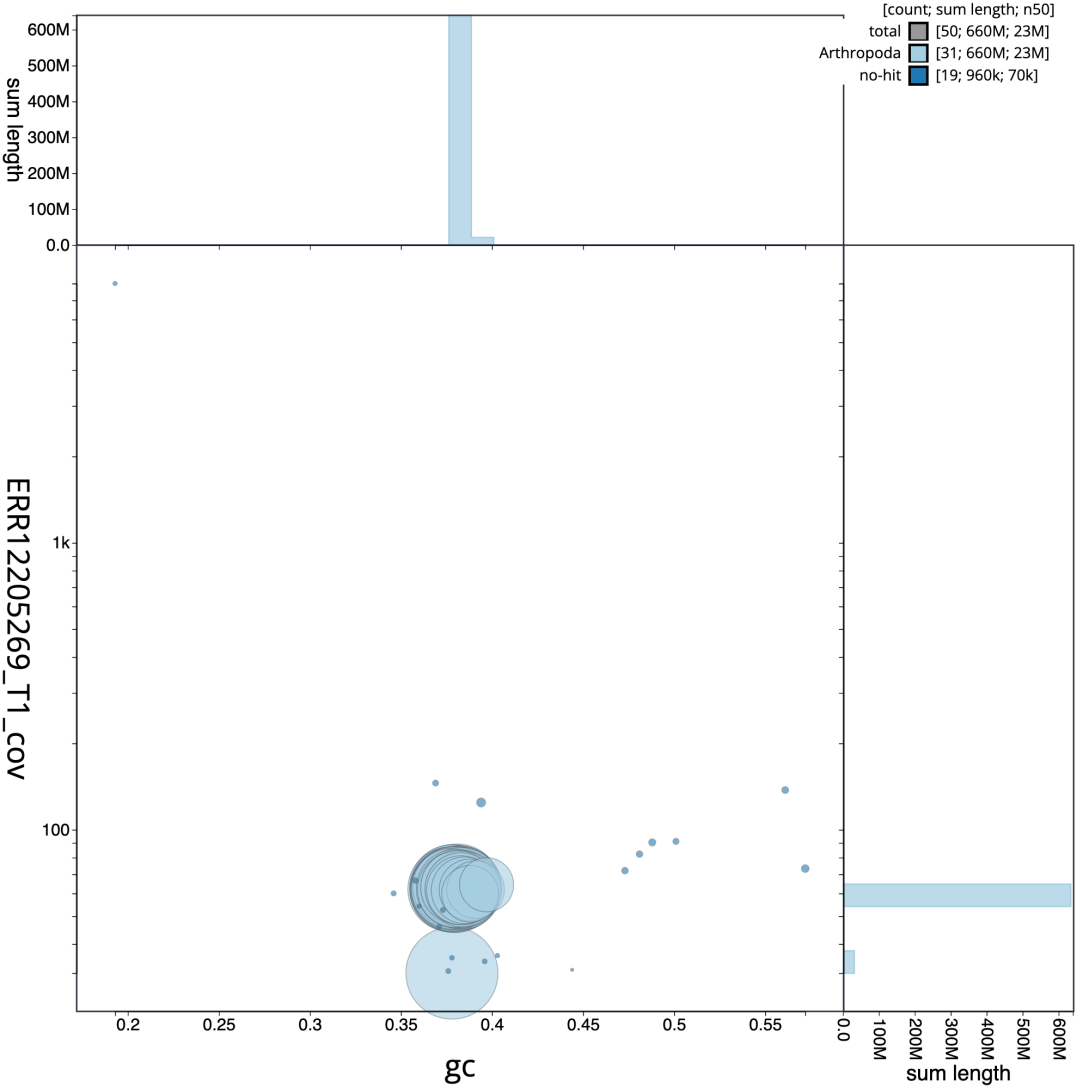
Genome assembly of *Luperina nickerlii*, ilLupNick1.1: BlobToolKit GC-coverage plot. Sequences are coloured by phylum. Circles are sized in proportion to sequence length. Histograms show the distribution of sequence length sum along each axis. An interactive version of this figure is available at https://blobtoolkit.genomehubs.org/view/Luperina_nickerlii/dataset/GCA_963855955.1/blob.

**Figure 4.**
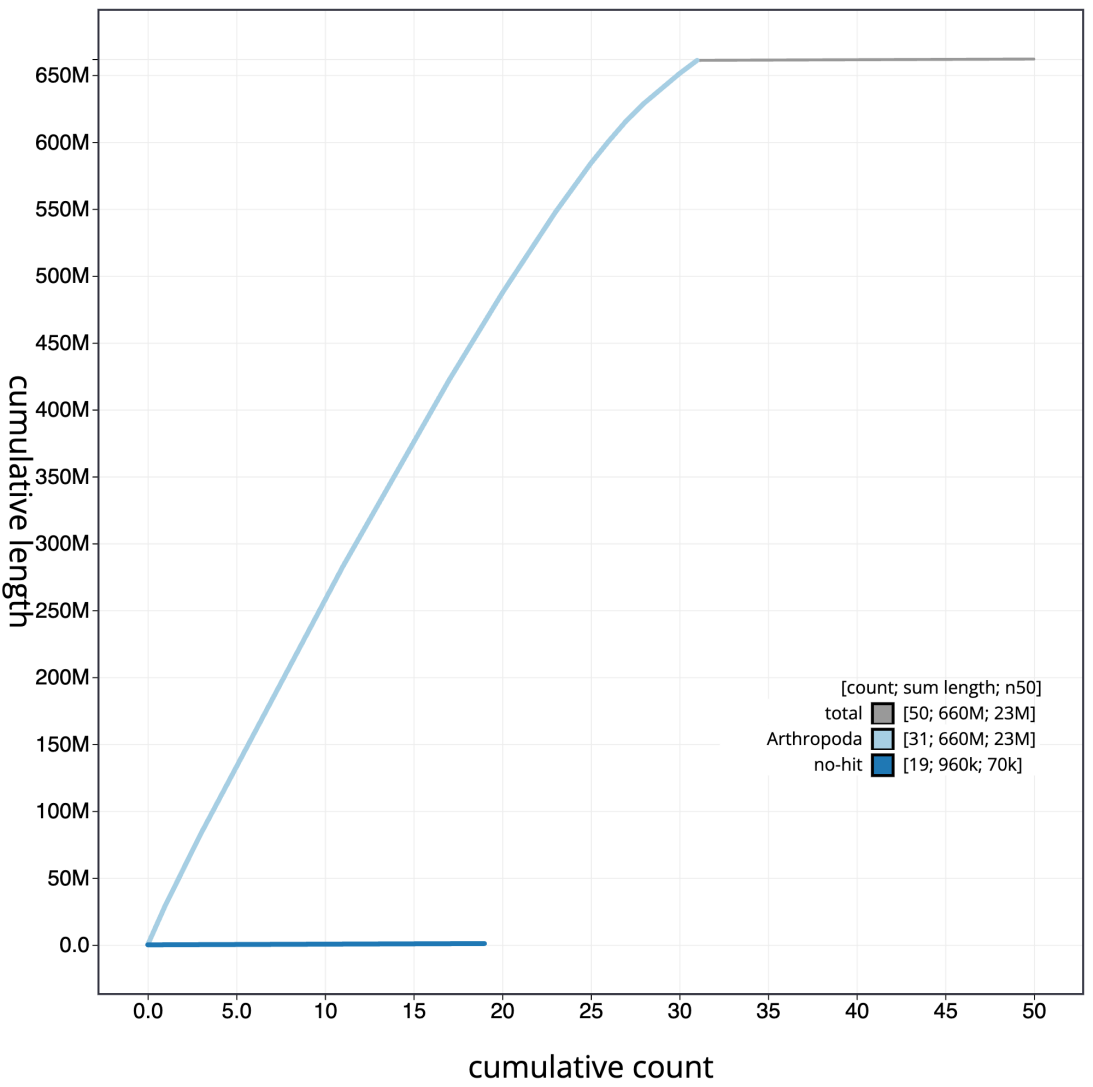
Genome assembly of *Luperina nickerlii* ilLupNick1.1: BlobToolKit cumulative sequence plot. The grey line shows cumulative length for all sequences. Coloured lines show cumulative lengths of sequences assigned to each phylum using the buscogenes taxrule. An interactive version of this figure is available at https://blobtoolkit.genomehubs.org/view/Luperina_nickerlii/dataset/GCA_963855955.1/cumulative.

**Figure 5.**
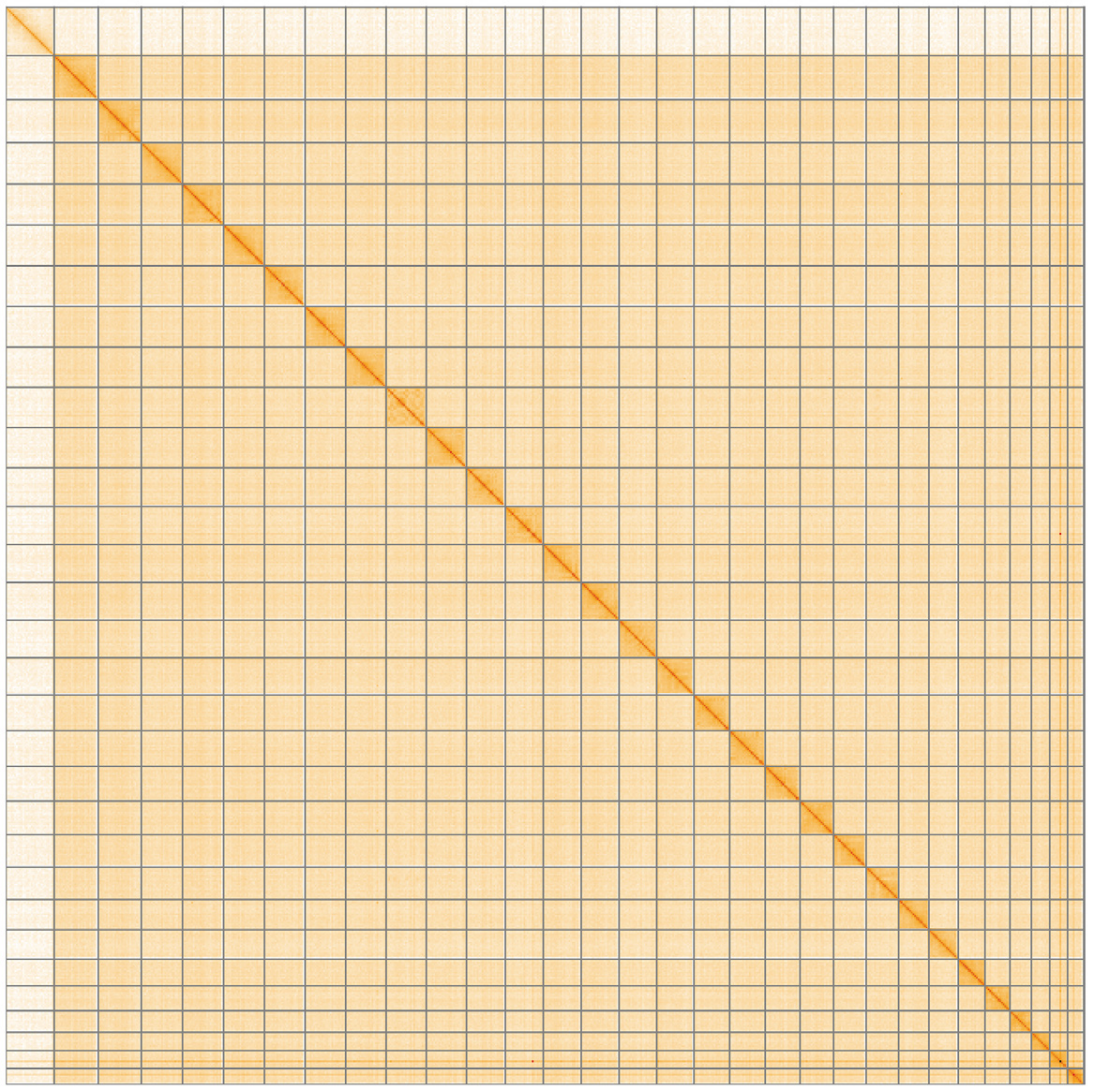
Genome assembly of *Luperina nickerlii* ilLupNick1.1: Hi-C contact map of the ilLupNick1.1 assembly, visualised using HiGlass. Chromosomes are shown in order of size from left to right and top to bottom. An interactive version of this figure may be viewed at https://genome-note-higlass.tol.sanger.ac.uk/l/?d=aPFVI1cUS36evra0niJFXQ.

The final assembly has a total length of 662.0 Mb in 49 sequence scaffolds with a scaffold N50 of 23.2 Mb ( Table 1). The snail plot in Figure 2 provides a summary of the assembly statistics, while the distribution of assembly scaffolds on GC proportion and coverage is shown in Figure 3. The cumulative assembly plot in Figure 4 shows curves for subsets of scaffolds assigned to different phyla. Most (99.85%) of the assembly sequence was assigned to 31 chromosomal-level scaffolds, representing 30 autosomes and the Z sex chromosome. Chromosome-scale scaffolds confirmed by the Hi-C data are named in order of size ( Figure 5; Table 2). Chromosome Z was assigned based on read coverage statistics. No W chromosome could be identified, and the species appears to be ZO.

**Table 1.** Genome data for *Luperina nickerlii*, ilLupNick1.1.

Project accession data		
Assembly identifier	ilLupNick1.1	
Species	*Luperina nickerlii*	
Specimen	ilLupNick1	
NCBI taxonomy ID	882792	
BioProject	PRJEB67619	
BioSample ID	Genome sequencing: SAMEA112975665 Hi-C scaffolding: SAMEA112975665	
Isolate information	ilLupNick1: thorax (genome sequence) ilLupNick1: thorax (Hi-C sequencing)	
Assembly metrics *		*Benchmark*
Consensus quality (QV)	65.7	*≥ 50*
*k*-mer completeness	100.0%	*≥ 95%*
BUSCO **	C:99.0%[S:98.7%,D:0.3%], F:0.2%,M:0.8%,n:5,286	*C ≥ 95%*
Percentage of assembly mapped to chromosomes	99.85%	*≥ 95%*
Sex chromosomes	Z	*localised homologous pairs*
Organelles	Mitochondrial genome: 15.47 kb	*complete single alleles*
Raw data accessions		
PacificBiosciences Revio	ERR12205269	
Hi-C Illumina	ERR12144004	
Genome assembly		
Assembly accession	GCA_963855955.1	
*Accession of alternate haplotype*	GCA_963855935.1	
Span (Mb)	662.0	
Number of contigs	173	
Contig N50 length (Mb)	8.1	
Number of scaffolds	49	
Scaffold N50 length (Mb)	23.2	
Longest scaffold (Mb)	29.64	

* Assembly metric benchmarks are adapted from column VGP-2020 of “Table 1: Proposed standards and metrics for defining genome assembly quality” from Rhie *et al.* (2021).

** BUSCO scores based on the lepidoptera_odb10 BUSCO set using version 5.4.3. C = complete [S = single copy, D = duplicated], F = fragmented, M = missing, n = number of orthologues in comparison. A full set of BUSCO scores is available at https://blobtoolkit.genomehubs.org/view/Luperina_nickerlii/dataset/GCA_963855955.1/busco.

**Table 2.** Chromosomal pseudomolecules in the genome assembly of *Luperina nickerlii*, ilLupNick1.

INSDC accession	Name	Length (Mb)	GC%
OY979663.1	1	27.0	38.0
OY979664.1	2	26.41	38.0
OY979665.1	3	25.3	38.0
OY979666.1	4	25.15	38.0
OY979667.1	5	25.06	38.0
OY979668.1	6	24.93	38.0
OY979669.1	7	24.91	37.5
OY979670.1	8	24.78	38.0
OY979671.1	9	24.65	38.0
OY979672.1	10	24.64	38.0
OY979673.1	11	23.81	38.0
OY979674.1	12	23.29	38.0
OY979675.1	13	23.24	38.0
OY979676.1	14	23.22	38.0
OY979677.1	15	23.06	38.0
OY979678.1	16	22.84	38.0
OY979679.1	17	21.9	38.0
OY979680.1	18	21.81	38.5
OY979681.1	19	21.36	38.0
OY979682.1	20	20.55	38.0
OY979683.1	21	19.99	38.0
OY979684.1	22	19.93	38.5
OY979685.1	23	18.58	38.0
OY979686.1	24	17.98	38.5
OY979687.1	25	16.38	38.5
OY979688.1	26	15.32	38.5
OY979689.1	27	13.12	39.0
OY979690.1	28	11.29	39.0
OY979691.1	29	10.95	39.0
OY979692.1	30	9.92	39.5
OY979662.1	Z	29.64	38.0
OY979693.1	MT	0.02	19.5

The LSU analysis of the *Luperina nickerlii* subsp. *leechi* genome shows even coverage of all expected BUSCO markers across all of the autosomes and the Z chromosome (Table S1 and Table S2). This even coverage of the autosomes supports the idea that this female is indeed ZO and that she does indeed lack a female associated W chromosome. It is also consistent with the absence of any potential neoW, whereby the W chromosome has joined onto one of the autosomes to form a neoW sex chromosome. We therefore assume that this female lacks a W chromosome entirely.

The observation that *Luperina nickerlii* subsp. *leechi* appears to be genuinely ZO is interesting, particularly given the unique ecology of this subspecies and its restriction to a single beach in a single county of the UK (Cornwall). However, we note that several other Noctuids have also been identified as being potentially ZO ( Traut & Marec, 1997) and that being ZO may therefore not be uncommon in this family of moths. Further work will examine if there is any potentially functional link between this subspecies being ZO and its peculiar ecology, starting with an examination of close relatives to see if the apparent loss of the W chromosome is confined to this subspecies alone.

While not fully phased, the assembly deposited is of one haplotype. Contigs corresponding to the second haplotype have also been deposited. The mitochondrial genome was also assembled and can be found as a contig within the multifasta file of the genome submission.

The estimated Quality Value (QV) of the final assembly is 65.7 with *k*-mer completeness of 100.0%, and the assembly has a BUSCO v5.4.3 completeness of 99.0% (single = 98.7%, duplicated = %), using the lepidoptera_odb10 reference set ( *n* = 5,286).

Metadata for specimens, barcode results, spectra estimates, sequencing runs, contaminants and pre-curation assembly statistics are given at https://links.tol.sanger.ac.uk/species/882792.

## Methods

### Sample acquisition and nucleic acid extraction

The genome was sequenced from a single female *Luperina nickerlii leechi* (ilLupNick1, Figure 2) collected from Loe Bar SSSI in Cornwall (latitude 50.07, longitude –5.29) after dark on 22 September 2022 by finding moths resting on the larval foodplant. The specimen was collected and identified by Adrian Spalding and then sent alive through the post by first class mail to Gavin Broad, Principal Curator (Hymenoptera) at the Natural History Museum, where it arrived the following day. The photograph of the specimen sequenced was provided by Gavin Broad.

The workflow for high molecular weight (HMW) DNA extraction at the Wellcome Sanger Institute (WSI) Tree of Life Core Laboratory includes a sequence of core procedures: sample preparation; sample homogenisation, DNA extraction, fragmentation, and clean-up. In sample preparation, the ilLupNick1 sample was weighed and dissected on dry ice ( Jay *et al.*, 2023). Tissue from thorax was homogenised using a PowerMasher II tissue disruptor ( Denton *et al.*, 2023a). HMW DNA was extracted in the WSI Scientific Operations core using the Automated MagAttract v2 protocol ( Oatley *et al.*, 2023). The DNA was sheared into an average fragment size of 12–20 kb in a Megaruptor 3 system with speed setting 31 ( Bates *et al.*, 2023). Sheared DNA was purified by solid-phase reversible immobilisation ( Strickland *et al.*, 2023): in brief, the method employs a 1.8X ratio of AMPure PB beads to sample to eliminate shorter fragments and concentrate the DNA. The concentration of the sheared and purified DNA was assessed using a Nanodrop spectrophotometer and Qubit Fluorometer and Qubit dsDNA High Sensitivity Assay kit. Fragment size distribution was evaluated by running the sample on the FemtoPulse system.

Protocols developed by the WSI Tree of Life laboratory are publicly available on protocols.io ( Denton *et al.*, 2023b).

### Sequencing

Pacific Biosciences HiFi circular consensus DNA sequencing libraries were constructed according to the manufacturers’ instructions. DNA sequencing was performed by the Scientific Operations core at the WSI on a Pacific Biosciences Revio (HiFi) instrument. Hi-C data were also generated from thorax tissue of ilLupNick1 using the Arima v2 kit. The Hi-C sequencing was performed using paired-end sequencing with a read length of 150 bp on the Illumina NovaSeq 6000 instrument.

### Genome assembly and curation

Assembly was carried out with Hifiasm ( Cheng *et al.*, 2021) and haplotypic duplication was identified and removed with purge_dups ( Guan *et al.*, 2020). The assembly was then scaffolded with Hi-C data ( Rao *et al.*, 2014) using YaHS ( Zhou *et al.*, 2023). The assembly was checked for contamination and corrected using the TreeVal pipeline ( Pointon *et al.*, 2023). Manual curation was performed using JBrowse2 ( Diesh *et al.*, 2023), HiGlass ( Kerpedjiev *et al.*, 2018) and PretextView ( Harry, 2022). The mitochondrial genome was assembled using MitoHiFi ( Uliano-Silva *et al.*, 2023), which runs MitoFinder ( Allio *et al.*, 2020) or MITOS ( Bernt *et al.*, 2013) and uses these annotations to select the final mitochondrial contig and to ensure the general quality of the sequence.

Following the assembly of all 31 autosomes and a single Z chromosome (see Results), we used LSU analysis ( Traut *et al.*, 2023) to examine the distribution of BUSCO markers across each chromosome. We wanted to test if the sequenced female was genuinely ZO and that it really lacks a W (female linked) sex chromosome. We also wanted to rule out the possibility that the W had become attached to one of the autosomes as a neoW sex chromosome (an autosome-W fusion) rather than having simply been lost.

### Evaluation of final assembly

The final assembly was post-processed and evaluated with the three Nextflow ( Di Tommaso *et al.*, 2017) DSL2 pipelines “sanger-tol/readmapping” ( Surana *et al.*, 2023a), “sanger-tol/genomenote” ( Surana *et al.*, 2023b), and “sanger-tol/blobtoolkit” ( Muffato *et al.*, 2024). The pipeline sanger-tol/readmapping aligns the Hi-C reads with bwa-mem2 ( Vasimuddin *et al.*, 2019) and combines the alignment files with SAMtools ( Danecek *et al.*, 2021). The sanger-tol/genomenote pipeline transforms the Hi-C alignments into a contact map with BEDTools ( Quinlan & Hall, 2010) and the Cooler tool suite ( Abdennur & Mirny, 2020), which is then visualised with HiGlass ( Kerpedjiev *et al.*, 2018). It also provides statistics about the assembly with the NCBI datasets ( Sayers *et al.*, 2024) report, computes *k*-mer completeness and QV consensus quality values with FastK and MerquryFK, and a completeness assessment with BUSCO ( Manni *et al.*, 2021).

The sanger-tol/blobtoolkit pipeline is a Nextflow port of the previous Snakemake Blobtoolkit pipeline ( Challis *et al.*, 2020). It aligns the PacBio reads with SAMtools and minimap2 ( Li, 2018) and generates coverage tracks for regions of fixed size. In parallel, it queries the GoaT database ( Challis *et al.*, 2023) to identify all matching BUSCO lineages to run BUSCO ( Manni *et al.*, 2021). For the three domain-level BUSCO lineage, the pipeline aligns the BUSCO genes to the Uniprot Reference Proteomes database ( Bateman *et al.*, 2023) with DIAMOND ( Buchfink *et al.*, 2021) blastp. The genome is also split into chunks according to the density of the BUSCO genes from the closest taxonomically lineage, and each chunk is aligned to the Uniprot Reference Proteomes database with DIAMOND blastx. Genome sequences that have no hit are then chunked with seqtk and aligned to the NT database with blastn ( Altschul *et al.*, 1990). All those outputs are combined with the blobtools suite into a blobdir for visualisation.

All three pipelines were developed using the nf-core tooling ( Ewels *et al.*, 2020), use MultiQC ( Ewels *et al.*, 2016), and make extensive use of the Conda package manager, the Bioconda initiative ( Grüning *et al.*, 2018), the Biocontainers infrastructure ( da Veiga Leprevost *et al.*, 2017), and the Docker ( Merkel, 2014) and Singularity ( Kurtzer *et al.*, 2017) containerisation solutions.

Table 3 contains a list of relevant software tool versions and sources.

**Table 3.** Software tools: versions and sources.

Software tool	Version	Source
BEDTools	2.30.0	https://github.com/arq5x/bedtools2
Blast	2.14.0	ftp://ftp.ncbi.nlm.nih.gov/blast/executables/blast+/
BlobToolKit	4.3.7	https://github.com/blobtoolkit/blobtoolkit
BUSCO	5.4.3 and 5.5.0	https://gitlab.com/ezlab/busco
bwa-mem2	2.2.1	https://github.com/bwa-mem2/bwa-mem2
Cooler	0.8.11	https://github.com/open2c/cooler
DIAMOND	2.1.8	https://github.com/bbuchfink/diamond
fasta_windows	0.2.4	https://github.com/tolkit/fasta_windows
FastK	427104ea91c78c3b8b8b49f1a7d6bbeaa869ba1c	https://github.com/thegenemyers/FASTK
GoaT CLI	0.2.5	https://github.com/genomehubs/goat-cli
Hifiasm	0.16.1-r375	https://github.com/chhylp123/hifiasm
HiGlass	44086069ee7d4d3f6f3f0012569789ec138f42b84 aa44357826c0b6753eb28de	https://github.com/higlass/higlass
MerquryFK	d00d98157618f4e8d1a9190026b19b471055b22e	https://github.com/thegenemyers/MERQURY.FK
MitoHiFi	2	https://github.com/marcelauliano/MitoHiFi
MultiQC	1.14, 1.17, and 1.18	https://github.com/MultiQC/MultiQC
NCBI Datasets	15.12.0	https://github.com/ncbi/datasets
Nextflow	23.04.0-5857	https://github.com/nextflow-io/nextflow
PretextView	0.2	https://github.com/wtsi-hpag/PretextView
purge_dups	1.2.3	https://github.com/dfguan/purge_dups
samtools	1.16.1, 1.17, and 1.18	https://github.com/samtools/samtools
sanger-tol/genomenote	1.1.1	https://github.com/sanger-tol/genomenote
sanger-tol/readmapping	1.2.1	https://github.com/sanger-tol/readmapping
Seqtk	1.3	https://github.com/lh3/seqtk
Singularity	3.9.0	https://github.com/sylabs/singularity
TreeVal	1.0.0	https://github.com/sanger-tol/treeval
YaHS	yahs-1.1.91eebc2	https://github.com/c-zhou/yahs

### Wellcome Sanger Institute – Legal and Governance

The materials that have contributed to this genome note have been supplied by a Darwin Tree of Life Partner. The submission of materials by a Darwin Tree of Life Partner is subject to the **‘Darwin Tree of Life Project Sampling Code of Practice’**, which can be found in full on the Darwin Tree of Life website here. By agreeing with and signing up to the Sampling Code of Practice, the Darwin Tree of Life Partner agrees they will meet the legal and ethical requirements and standards set out within this document in respect of all samples acquired for, and supplied to, the Darwin Tree of Life Project.

Further, the Wellcome Sanger Institute employs a process whereby due diligence is carried out proportionate to the nature of the materials themselves, and the circumstances under which they have been/are to be collected and provided for use. The purpose of this is to address and mitigate any potential legal and/or ethical implications of receipt and use of the materials as part of the research project, and to ensure that in doing so we align with best practice wherever possible. The overarching areas of consideration are:

• Ethical review of provenance and sourcing of the material

• Legality of collection, transfer and use (national and international) 

Each transfer of samples is further undertaken according to a Research Collaboration Agreement or Material Transfer Agreement entered into by the Darwin Tree of Life Partner, Genome Research Limited (operating as the Wellcome Sanger Institute), and in some circumstances other Darwin Tree of Life collaborators.

## Data Availability

European Nucleotide Archive: *Luperina nickerlii* (sandhill rustic). Accession number PRJEB67619; https://identifiers.org/ena.embl/PRJEB67619 ( Wellcome Sanger Institute, 2023). The genome sequence is released openly for reuse. The *Luperina nickerlii* genome sequencing initiative is part of the Darwin Tree of Life (DToL) project. All raw sequence data and the assembly have been deposited in INSDC databases. The genome will be annotated using available RNA-Seq data and presented through the Ensembl pipeline at the European Bioinformatics Institute. Raw data and assembly accession identifiers are reported in Table 1.

## Author information

Members of the Natural History Museum Genome Acquisition Lab are listed here: https://doi.org/10.5281/zenodo.7139035.

Members of the Darwin Tree of Life Barcoding collective are listed here: https://doi.org/10.5281/zenodo.4893703.

Members of the Wellcome Sanger Institute Tree of Life Management, Samples and Laboratory team are listed here: https://doi.org/10.5281/zenodo.10066175.

Members of Wellcome Sanger Institute Scientific Operations: Sequencing Operations are listed here: https://doi.org/10.5281/zenodo.10043364.

Members of the Wellcome Sanger Institute Tree of Life Core Informatics team are listed here: https://doi.org/10.5281/zenodo.10066637.

Members of the Tree of Life Core Informatics collective are listed here: https://doi.org/10.5281/zenodo.5013541.

Members of the Darwin Tree of Life Consortium are listed here: https://doi.org/10.5281/zenodo.4783558.

## Supplementary information

Supplementary Tables 1 and 2 are given at https://doi.org/10.5281/zenodo.11494741.
