# Supplementary material for: The genome sequence of the Sandhill Rustic moth Luperina nickerlii (Freyer, 1845) subspecies leechi Goater, 1976
Source: Wellcome Open Res. 2024 Jul 17;9:382. [Version 1] doi: 10.12688/wellcomeopenres.22623.1 (PMC11499737; doi:10.12688/wellcomeopenres.22623.1)
Supplement: Peer review history

# Reviewer report: Reviewer response for version 1

**DOI:** 10.21956/wellcomeopenres.24923.r100706

**Published:** 2024-10-23

**Authors:** Ken Sahara

**Affiliations:** 1 Iwate University, Morioka, Japan

Genome sequence data of insects will largely contribute to understand species and ecological divergences of lives. *Luperina nickerlii*, has several subspecies. The target moth in this study, *L. nickerlii leechi *exists in a restricted area with limiting population size and is potentially under threat from habitat loss and climate change. Recording the genome data of such specimen is worth for future comparison not only within subspecies but also among moths and or insects.    

**Minor comment:**

 M&M Sample acquisition and nucleic acid extraction:

 Delete “. io” in the last paragraph.

Are sufficient details of methods and materials provided to allow replication by others?

Yes

Is the rationale for creating the dataset(s) clearly described?

Yes

Are the datasets clearly presented in a useable and accessible format?

Yes

Are the protocols appropriate and is the work technically sound?

Yes

Reviewer Expertise:

NA

I confirm that I have read this submission and believe that I have an appropriate level of expertise to confirm that it is of an acceptable scientific standard.

# Reviewer report: Reviewer response for version 1

**DOI:** 10.21956/wellcomeopenres.24923.r99578

**Published:** 2024-10-16

**Authors:** Muhammad Asghar Hassan

**Affiliations:** 1 Guizhou University, Guiyang, China

I am pleased to accept the current manuscript on the genome of the Sandhill Rustic moth, *Luperina nickerlii* (Freyer, 1845) subspecies leechi Goater, 1976. The authors have provided comprehensive details on this species and its subspecies, along with well-presented figures. However, I would like to suggest the following corrections for the revised version:

1. There should be a comma between the common name and the scientific name and add the higher classification in bracket at the of the title rather than in abstract.
2. It is better to retain only the common name after the scientific name, and transfer Arthopoda and Insecta in keywords, as Lepidoptera and Noctuidae in the title.
3. The author name for this species should be placed in brackets. Replace “ *nickerlii* Freyer, 1845” with “ *nickerlii* (Freyer, 1845)”
4. Why are there no references for these statements? I believe the revised version should include valid references to support them.

Are sufficient details of methods and materials provided to allow replication by others?

Yes

Is the rationale for creating the dataset(s) clearly described?

Yes

Are the datasets clearly presented in a useable and accessible format?

Yes

Are the protocols appropriate and is the work technically sound?

Yes

Reviewer Expertise:

Insect Taxonomy, Molecular Systematics, DNA Barcoding, Mitochondrial Genome

I confirm that I have read this submission and believe that I have an appropriate level of expertise to confirm that it is of an acceptable scientific standard.

# Response

**Published:** 2024-10-17

**Authors:** Tree of Life Team Sanger

**Affiliations:** Wellcome Trust Sanger Institute, UK

Thank you for reviewing this data note. We believe that points 1 and 2 of your comments are matters of personal preference, and have the following responses to points 3 and 4.  3. The author name for this species should be placed in brackets. Replace “ *nickerlii* Freyer, 1845” with “ *nickerlii* (Freyer, 1845).

 Placing the taxonomic authority in brackets would be incorrect in this case. The author name is not in parentheses because Freyer, 1845 is the original authority for *nickerlii*. Parentheses are used when the species has been moved to a different genus since the original description.

 4. Why are there no references for these statements? I believe the revised version should include valid references to support them.

 We would like to point out that the Sandhill Rustic is extensively covered in the reference supplied (Spalding, 2015). The writers of the background section are authorities on this moth.
